# Global Nanotribology Research Output (1996–2010): A Scientometric Analysis

**DOI:** 10.1371/journal.pone.0081094

**Published:** 2013-12-05

**Authors:** Bakthavachalam Elango, Periyaswamy Rajendran, Lutz Bornmann

**Affiliations:** 1 Library, IFET College of Engineering, Villupuram, Tamilnadu, India; 2 University Library, SRM University, Kattangulathur, Tamilnadu, India; 3 Division for Science and Innovation Studies, Administrative Headquarters of the Max Planck Society, Munich, Germany; Katholieke Universiteit Leuven, Belgium

## Abstract

This study aims to assess the nanotribology research output at global level using scientometric tools. The SCOPUS database was used to retrieve records related to the nanotribology research for the period 1996–2010. Publications were counted on a fractional basis. The level of collaboration and its citation impact were examined. The performance of the most productive countries, institutes and most preferred journals is assessed. Various visualization tools such as the Sci^2^ tool and Ucinet were employed. The USA ranked top in terms of number of publications, citations per paper and h-index, while Switzerland published a higher percentage of international collaborative papers. The most productive institution was Tsinghua University followed by Ohio State University and Lanzhou Institute of Chemical Physics, CAS. The most preferred journals were *Tribology Letters*, *Wear* and *Journal of Japanese Society of Tribologists*. The result of author keywords analysis reveals that Molecular Dynamics, MEMS, Hard Disk and Diamond like Carbon are major research topics.

## Introduction

Nanotribology is a branch of tribology and was coined by Krim, Solina and Chiarello [1] in a paper entitled “Nanotribology of a Kr monolayer”. Nanotribology deals with tribological phenomena occurring at sub-micron or smaller length scales; tribology is the science and technology of interactive surfaces in relative motion, which include the studies of friction, lubrication and wear [2]. The difference between tribology and nanotribology is comparable to the Newtonian physics and quantum physics [3]. Nanotribology is a highly interdisciplinary field where tribologists, physicists, chemists, material scientists, micro/nano- and miniature system engineers are jointly developing theories, simulations, and final applications that benefit society [4]. Today nanotribology is one of the most important mechanical technologies and it uses many new instruments such as the surface force apparatus, atomic force microscope, friction force microscope and scanning tunnel microscope [5]. The emergence of micro/nanotribology and atomic force microscopy based techniques has provided researchers with a viable approach to addressing tribological problems [6], [7]. Nanotribological studies are mostly fundamental in nature and the results are applied in the MEMS, in HDD technologies as well as in nanotechnologies [8]. Nanotribology has brought the scale of interest, familiar in physics and chemistry, to the level of an engineering phenomenon. The future of nanotechnology depends on advancements in nanotribology, which has wide applications ranging from health care to energy conversion and storage, and micro craft space exploration [9]. Applications include improving car engine lubrications, biolubrication in hip joints and cosmetics, shrinking devices to micrometer and nanometer scales to manufacture nanoscale machines, and expanding the range of temperatures, speeds, and chemical environments to the extreme conditions where devices operate [10].

Evaluating research fields like nanotribology using scientometric techniques is useful in determining, for example, the citation impact of contributing authors and institutions [11]. Findings from these investigations can help researchers to realize the breadth of research in the field and establish possible future research directions [12]. Bibliometric/scientometric studies have been carried out in the past in various research fields ranging from science to engineering and medicine (see a selection in Table 1). The aim of the present study is: (1) to examine the publications pattern of nanotribology research output at global level; (2) to analyse the publications pattern and impact of the most producing countries; (3) to examine the productivity and impact of the most publishing institutions; (4) to analyse the pattern of authorship and prolific authors; (5) to examine the impact of the most preferred journals; (6) to analyse the characteristics of highly cited papers; (7) to analyse the keywords appended by the authors; (8) to visualize the co-authorship network among the authors and collaboration network among the top ten most productive countries.

**Table 1:** Recent bibliometric/scientometric studies.

Author(s) and year	Research field
Modak and Giridhar (2008) [13]	Chemical Engineering
Tsay (2008) [14]	Hydrogen Energy
Ortiz et al (2009) [15]	Cancer
Sun, Wang and Ho (2012) [16]	Estuary Pollution
Yu et al (2012) [17]	Photosynthesis
Dong et al (2012) [18]	Solar Power
Wang et al (2013) [19]	GPS
Zhou and Wang (2013) [20]	Laparoscopy
Fu, Wang and Ho (2013) [21]	Drinking Water
Kademani et al (2013) [22]	Materials Science

## Materials and Methods

SCOPUS (Elsevier) was used to retrieve the records related to nanotribology research for the period 1996–2010. The following keywords were used in the combined field of title, abstract and keywords: nanotribo* OR microtribo* OR nano-tribo* OR micro-tribo* OR {*nano tribology*} OR {*micro tribology*}. The search was carried out on 11/15/2012 and refined to restrict the literature to articles, conference papers and reviews [23]. Bibliographic details related to nanotribology research are available from 1974 in SCOPUS. Since SCOPUS does not have complete citation information for papers published before 1996 [24], the present study was confined to 1996–2010. Self-citations (of authors, institutions, etc.) have not been excluded from the analyses.

The retrieved data were exported to MS-Excel. 29 records were deleted where the information related to author and affiliation was not available. We proceeded with 1321 papers related to nanotribology research during the period 1996–2010. Manual coding was done for the number of authors, country of origin and affiliation of authors. The fractional counting method was applied to give credit to all the contributing authors, institutes and countries [25]. Institutional affiliations and author names were unified manually.

### Tools and Techniques Employed

The impact of research is measured by citations [23]. Since we measure the citation impact within one field (nanotribology) only, we did not apply field-normalized indicators (such as the relative citation rate). Citations per publication (CPP) can be used to assess the impact of publications for publication years, countries, institutes and authors. The formula of CPP is

CPP  =  Total Citations / Total Papers

Both the output and impact of the publications of the most productive countries, institutes and journals are measured using the h-index. Hirsch [26] proposed the h-index as an alternative to standard bibliometric indicators for single scientists; it is defined as follows:

A scientist has index h if h of his or her N_p_ papers have at least h citations each and other papers (N_p_–h) have ≤h citations each.

UCinet [32] is used to generate a collaborations network among the top ten most productive countries in nanotribology research. To construct the collaboration network map, the following steps were taken.

Among the various available methods to calculate the h-index, Ye [27] found that the Glänzel-Schubert [28] model was better than the Hirsch and Egghe-Rousseau [29] models to estimate the h-index of a publication set. The difference between these models is that the original h-index model links only total citations and the Egghe-Rousseau model links only total publications, whereas the Glänzel-Schubert model incorporates the total citations as well as total publications (Table 2). Since its introduction in 2005, the h-index has been applied not only to single scientists, but also to research groups [30] and countries [31]. For example, Fu, Wang and Ho [21] applied the h-index to countries, institutes and journals.

**Table 2:** Various models of the h-index.

Model	Equation	Description
Hirsch	h = √(C/a)	C = total citations, a is a constant ranging from 3 to 5
Egghe-Rousseau	h = P^1/α^	P = total publications, α >1 is Lotka’s exponent
Glänzel-Schubert	h = cP^1/3^(CPP)^2/3^	c is a constant (0.9 for journals and 1 for other units), P = total publications, CPP = citations per publication

Step 1 – A matrix was developed using the number of papers collaborated on by the countries with each other country among the top ten in Excel matrix editor.

Step 2 – Collaboration network was visualized with Netdraw [33]

**Figure 1:**
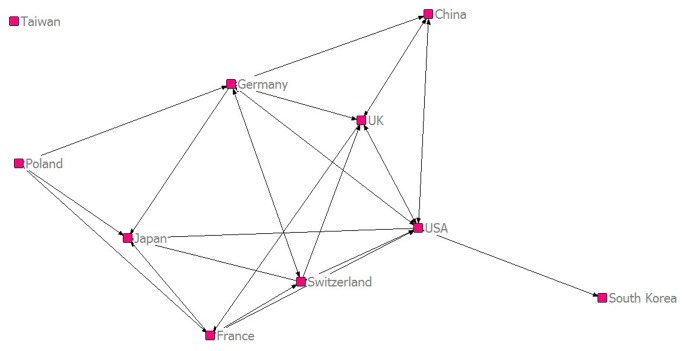
Collaboration network among the top ten most productive countries.

Step 3 – Colours of the nodes were changed (Figure 1).

The Sci^2^ tool [34] is used to generate a co-author network map among the authors of nanotribology research. To construct the visualization map, the following steps were taken.

Step 1 – Load CSV file was selected

Step 2 – Extract co-author network was selected (file format: SCOPUS)

Step 3 – Network Analysis Tool kit was selected

**Figure 2:**
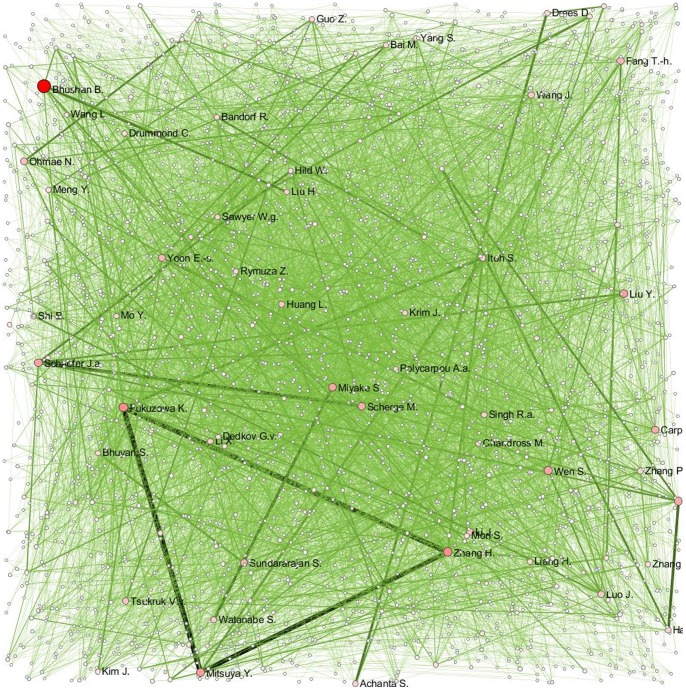
Co-authorship Network. Each node represents one author and the size of the node denotes the number of papers. The thickness of interconnecting lines (edges) denotes the number of co-authored papers between the authors.

Step 4 – GUESS [35] was selected and default random layout used (Figure 2)

Step 5 – Extract K-core was selected (Unweighted and Directed)

**Figure 3:**
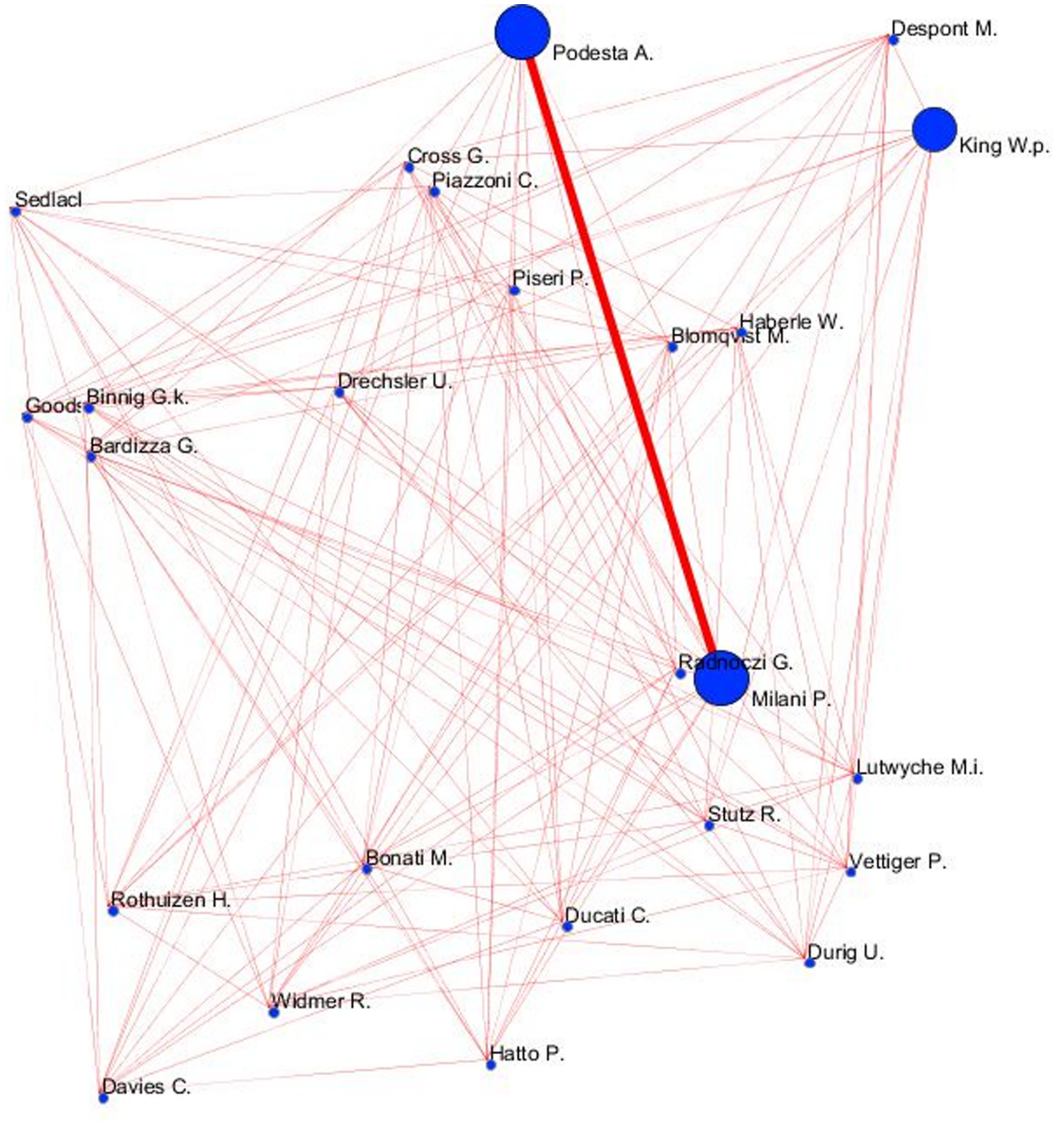
K-core of co-author network (k = 10). The size of the node denotes the number of papers and the thickness of interconnecting lines (edges) denotes the number of co-authored papers between the authors. Authors having 10 or more co-author links during the study period have been visualized.

Step 6 – GUESS was selected and default random layout used (Figure 3).

## Results

### Growth of Publications and Citations

Table 3 provides the following general characteristics about nanotribology research output for the period 1996–2010: annual output, average number of authors, number of citations received, citations per publication, percentage of cited documents and citations per publication per year. Nanotribology research increased tremendously from 34 papers in 1996 to 161 papers in 2010, with an average of 88 papers per year. The highest number of papers was published in the year 2008, at 177, and the lowest in 1997, at 17. The average number of authors per paper increased from 2.41 in 1996 to 3.66 in 2010, with an average of 3.52 authors per paper. This increase reflects the increasing trend of (international) collaboration in this research field. Out of total publications, 70% of papers received one or more citations. Overall, 11913 citations were received by 1321 papers, with an average of 9 per paper; citations per paper per year were 1.36.

**Table 3:** Year-wise output, average authors and citations.

Year	TP	AU	AU/P	TC	CPP	Cited	% cited	CPPY
1996	34	82	2.41	876	25.76	23	67.65	1.61
1997	17	48	2.82	445	26.18	15	88.24	1.75
1998	55	174	3.16	814	14.80	44	80.00	1.06
1999	38	126	3.32	604	15.89	28	73.68	1.22
2000	71	228	3.21	822	11.58	49	69.01	0.96
2001	67	222	3.31	766	11.43	51	76.12	1.04
2002	48	180	3.75	492	10.25	38	79.17	1.03
2003	67	252	3.76	861	12.85	55	82.09	1.43
2004	99	323	3.26	858	8.67	66	66.67	1.08
2005	139	511	3.68	1319	9.49	92	66.19	1.36
2006	115	447	3.89	948	8.24	84	73.04	1.37
2007	101	372	3.68	1129	11.18	79	78.22	2.24
2008	177	615	3.47	947	5.35	104	58.76	1.34
2009	132	486	3.68	524	3.97	86	65.15	1.32
2010	161	590	3.66	508	3.16	114	70.81	1.58
Total	1321	4656		11913		928		
Mean			3.52		9.02		70.25	1.36

TP = total papers, AU = number of authors, AU/P = avg. authors per paper, TC = total citations, CPP = citations per paper, CPPY = citations per paper per year.

### Level of Collaboration

Collaboration type is determined by the author affiliations of each paper as follows (see Table 4): (i) Single-authored publications; (ii) Single institute publications with author affiliations from the same institution; (iii) Inter-institutionally collaborative publications with different author affiliations within the same country; (iv) Single country publications with author affiliations from the same country; (v) International collaborative publications with author affiliations from different countries. Single-authored publications are very nominal (11%) and multi-authored publications dominate the research field. The level of collaboration and its citation impact of nanotribology research output during 1996–2010 are presented in Table 4, which indicates that the publications contributed with international collaboration had the highest impact, with an average CPP of 11.71, while single institute publications had the lowest impact, at 8.47.

**Table 4:** Level of collaboration and citation impact.

Level	TP	% of TP	TC	CPP
Without collaboration(single-authored)	141	10.67	1247	8.84
Collaboration withinternational institutions	161	12.19	1885	11.71
Collaboration withanother institution	314	23.77	2811	8.95
Collaboration withinthe same institution	705	53.37	5970	8.47
Total	1321		11913	

TP = total papers, TC = total citations, CPP = citations per paper.

### Most Productive Countries

Forty four countries were involved in the total research output (n = 1321) on nanotribology during 1996–2010. About 84% of total publications were contributed by the top ten most productive countries, which indicate that the researchers from these countries were involved more in this research field compared to other countries and 85% of total citations were received by the publications contributed by these top ten countries. G7 countries (USA, UK, France, Germany, Italy, Canada, and Japan) contributed 58% of total publications during the study period and five of the G7 countries are ranked among the top ten countries. The domination of the G7 countries has occurred in most of the research fields [18]. The top ten most productive countries on nanotribology research during the period 1996–2010 are ranked and listed in Table 5. The rankings are based on total publications including single country papers, international collaborative papers, and h-index. The USA published the most papers (n = 330.67) and is ranked top in terms of single country papers, international collaborative papers and received the highest h-index of 44. China ranked second in terms of total publications and single country papers. However, China ranked third in terms of international collaborative papers and fourth in h-index (h = 17). Germany ranked second in terms of h-index, while it is ranked fourth in terms of total publications.

**Table 5:** Top ten most productive countries and their rank.

Country	TP	R(TP %)	R(SCP %)	R(ICP %)	% C	CPP	R(h)
USA	330.67	1 (25.03)	1 (25.97)	1 (18.43)	8.97	15.91	1 (44)
China	252.5	2 (19.11)	2 (20.28)	3 (10.87)	6.93	4.38	4 (17)
Japan	201	3 (15.22)	3 (17.00)	12 (2.48)	1.99	4.54	5 (16)
Germany	108.5	4 (8.21)	4 (7.68)	2 (12.11)	17.97	8.28	2 (20)
UK	59	5 (4.47)	5 (3.88)	4 (8.70)	23.73	10.17	3 (18)
South Korea	43.33	6 (3.28)	6 (3.36)	11 (2.69)	9.99	11.57	3 (18)
France	38.33	7 (2.9)	7 (2.76)	7 (3.93)	16.51	10.04	5 (16)
Taiwan	32.67	8 (2.47)	8 (2.76)	25 (0.42)	2.05	6.00	7 (11)
Switzerland	29.67	9 (2.25)	9 (1.47)	5 (7.87)	42.7	10.28	6 (15)
Poland	20.5	10 (1.55)	10 (1.38)	10 (2.80)	21.95	2.17	8 (5)

TP = total papers, R = rank, SCP = single country papers, ICP = international collaborative papers, % C = percent of ICP in its total papers, CPP = citations per paper, h = h-index.

Figure 1 provides the collaboration network (Ucinet) among the top ten countries. It can be seen that collaborations among the top ten countries were frequent. The exception is Taiwan, which is not integrated in this network of the other top ten countries.

### Top Ten Most Productive Institutes

There were around 680 institutions worldwide involved in the 1321 publications during 1996–2010. Of the total of 1321 publications, 846 (64%) were single institute publications and the remaining 475 (36%) were inter-institutionally collaborated publications. The performance of the top ten most productive institutes was examined and is presented in Table 6. The top ten institutes published 21% of all papers. Even though the UK, France, Taiwan, Switzerland and Poland were ranked among the top ten countries, they had no institute among the top ten in Table 6. Of the top 10 most productive institutes, four are in the USA, two each in China and Japan, and one each in South Korea and Germany. Tsinghua University of China ranked top in terms of number of publications, but had the second-lowest value for the h-index. Ohio State University of the USA ranked second in terms of number of publications and had the highest h-index of 36. Among the top most productive institutes, Ilmenau University of Technology, Germany, and University of California, USA published the highest percentages of papers with inter-institutional collaboration in their total publications, at 46% and 37% respectively. Ohio State University, Iowa State University, University of California and University of Illinois originate from the USA and produced 33% of their country’s total output. Apart from the academic and research institutions, the following corporate bodies (among others) were involved in nanotribology research: Hitachi Ltd., Micro Materials Ltd., Falex Tribology NV, Caterpillar Inc., Ford Motor Company, Kao Corporation, and IBM.

**Table 6:** Most productive institutes.

Institute	TP (R)	IICP	% IICP	CPP	h
Tsinghua University (China)	50.50 (1)	9.49	19	3.17	8
Ohio State University (USA)	50.42 (2)	5.42	11	29.99	36
Lanzhou Institute of Chemical Physics, CAS (China)	31.33 (3)	5.33	17	10.18	15
Nagoya University (Japan)	29.33 (4)	2.33	8	4.45	8
Iowa State University (USA)	22.50 (5)	4.5	20	7.87	11
Ilmenau University of Technology (Germany)	22.24 (6)	10.24	46	10.06	13
University of California (USA)	20.74 (7)	7.74	37	34.32	29
Nippon Institute of Technology (Japan)	19 (8)	2	11	2.55	5
Korea Institute of Science and Technology (South Korea)	17.83 (9)	5.83	33	13.54	15
University of Illinois (USA)	15.58 (10)	2.58	17	12.15	13

TP = total papers, R = rank, IICP = inter-institutionally collaborated papers, % IICP = percent of IICP in its total papers, CPP = citations per paper, h = h-index.

### Core Journals

Journals which published at least 20 papers related to nanotribology research during 1996–2010 are listed in Table 7. Ten journals published 20 or more papers and these journals published 33% of all papers. About 45% of all citations were received by the papers published in the top ten journals. *Tribology Letters* (n = 130) was the top journal by publication output, followed by *Wear* (n = 66) and the *Journal of Japanese Society of Tribologists* (n = 51). *Langmuir* had the highest impact with CPP of 29.63 and *Wear* received the highest h-index of 32 among the top ten journals. The *Journal of Japanese Society of Tribologists* ranked third in terms of total papers and it received the second-lowest h-index of 3. These core journals are in the subject areas of physics, materials science and engineering.

**Table 7:** Top ten most preferred journals.

Journal	TP (R)	CPP	h
*Tribology Letters*	130 (1)	12.38	27
*Wear*	66 (2)	22.06	32
*Journal of Japanese Society of Tribologists*	51 (3)	0.61	3
*Tribology International*	42 (4)	9.79	16
*Surface and Coatings Technology*	35 (5)	11.14	16
*Langmuir*	30 (6)	29.63	30
*Mocaxue Xuebao/Tribology*	26 (7)	2.50	5
*Japanese Journal of Tribology*	22 (8)	0.09	1
*Applied Surface Science*	21 (9)	8.62	12
*Thin Solid Films*	21 (9)	18.14	19

TP = total papers, R = rank, CPP = citations per paper, h = h-index.

### Most Highly Cited Papers

The characteristics of highly cited papers (the 1% most highly cited papers) are listed in Table 8 among the papers related to nanotribology research during 1996–2010. Citations received by the 13 top cited papers accumulated to 1503 (12%) of all citations. Of the 13 papers, 3 have a single author while the others have more than one author. Twelve most cited papers are single country papers and originated from the USA (10), UK (1) and Belgium (1). The most cited papers were published in ten different journals. The top cited paper was “Surface engineering and microtribology for microelectromechanical systems” authored by Komvopoulos K. from the USA and published in *Wear* in 1996. In this paper, the analysis of various surface micromechanisms, such as solid bridging, liquid meniscus formation, van der waals force, and electrostatic charging and the significance of surface roughness and material properties are emphasized. *Wear* comes under the subject categories of engineering and materials science.

**Table 8:** Characteristics of highly cited papers.

Paper	TC	CPY	Country of origin
Komvopoulos K. (1996). Surface engineering and microtribology for microelectromechanical systems.*Wear,* 200 (1–2): 305–327.	263	16.44	USA
Xu S., Miller S., Laibinis P. E., Liu G. - Y. (1999). Fabrication of nanometer scale patterns within self-assembledmonolayers by nanografting. *Langmuir,* 15 (21): 7244–7251.	163	12.54	USA
Sheehan P. E., Lieber C. M. (1996). Nanotribology and nanofabrication of MoO3 structures by atomic forcemicroscopy. *Science,* 272 (5265): 1158–1161.	134	8.38	USA
Liu H., Bhushan B. (2003). Nanotribological characterization of molecularly thick lubricant films for applicationsto MEMS/NEMS by AFM. *Ultramicroscopy,* 97 (1–4): 321–340.	123	13.67	USA
Johnson K. L. (1997). Adhesion and friction between a smooth elastic spherical asperity and a plane surface.*Proceedings of the Royal Society A: Mathematical, Physical and Engineering Sciences,* 453 (1956): 163–179.	123	8.20	UK
Enachescu M., Van Den Oetelaar R. J. A., Carpick R. W., Ogletree D. F., Flipse C. F. J., Salmeron M. (1998).Atomic force microscopy study of an ideally hard contact: The diamond(111)/tungsten carbide interface.*Physical Review Letters,* 81 (9): 1877–1880.	98	7.00	USA, Netherlands
Bhushan B., Liu H. (2001). Nanotribological properties and mechanisms of alkylthiol and biphenyl thiolself-assembled monolayers studied by AFM. *Physical Review B - Condensed Matter and Materials Physics,*63 (24): 5412-1-5412-11.	96	8.73	USA
Liu H., Bhushan B. (2002). Investigation of nanotribological properties of self-assembled monolayerswith alkyl and biphenyl spacer chains (Invited). *Ultramicroscopy,* 91 (1–4): 185–202.	89	8.90	USA
Szlufarska I., Chandross M., Carpick R. W. (2008). Recent advances in single-asperity nanotribology.*Journal of Physics D: Applied Physics,* 41 (12): 123001.	88	22.00	USA
Kim S. H., Asay D. B., Dugger M. T. (2007). Nanotribology and MEMS. *Nano Today,* 2 (5): 22–29.	85	17.00	USA
Liu E., Blanpain B., Celis J. P. (1996). Calibration procedures for frictional measurements with a lateralforce microscope. *Wear*, 192 (1–2): 141–150.	83	5.19	Belgium
Tsukruk V. V., Everson M. P., Lander L. M., Brittain W. J. (1996). Nanotribological properties of compositemolecular films: C60 anchored to a self-assembled monolayer. *Langmuir*, 12 (16): 3905–3911.	80	5.00	USA
Bhushan B. (2007). Nanotribology and nanomechanics of MEMS/NEMS and BioMEMS/BioNEMS materialsand devices. *Microelectronic Engineering*, 84 (3): 387–412.	78	15.60	USA

TC = total citations, CPY = citations per year.

### Authorship Pattern

Authorships vary from single to a maximum of 13 authors in the field of nanotribology research during the period 1996–2010. It can be observed from Table 9 that the highest percentage of contributions (22%) was made by three authors, followed by four authors and two authors, with 21% and 20% respectively. There was one paper with the highest number of authors (n = 13) which received the highest CPP of 32 among the authorships. Around 80% of the publications were contributed within a range of authors between 2 and 5. Only 11% of publications were contributed by a single author.

**Table 9:** Authorship pattern and its citation impact.

# authors	# papers	%	TC	CPP
1	141	11	1247	8.84
2	266	20	2799	10.52
3	294	22	2413	8.21
4	272	21	2387	8.78
5	195	15	1443	7.40
6	90	7	1054	11.71
7	30	2	290	9.67
8	18	1	186	10.33
9	11	1	39	3.55
10	2	0	18	9.00
12	1	0	5	5.00
13	1	0	32	32.00
Total	1321	100	11913	

TC = total citations, CPP = citations per paper.

### Co-authorship Network

There were 2581 authors involved in the total of 1321 papers in the field of nanotribology during 1996–2010. This shows that the research in this area was well diffused with many authors. The co-authorship network in Figure 2 was visualized using the Sci^2^ tool. Each node represents one author and the size of the node denotes the number of papers. The thickness of interconnecting lines denotes the number of co-authored papers. Authors with a significant number of papers can be identified from the visualization map, which indicates that the trio of Fukuzawa K., Zhang H., and Mitsuya K. co-authored the most during the study period. Apart from this trio, there exist significant links between Schaefer J. A. and Scherge M., Miyake S. and Watanabe S., Bhushan B. and Liu H. as well as Dress D. and Achanta S. The visualization map indicates that most author pairs have not co-authored with the same intensity as this trio of Fukuzawa K., Zhang H., and Mitsuya K.

Table 10 provides the general properties of the co-authorship network, which indicates that only 0.18% of all possible edges (co-authorships) are present during the study period. This percentage indicates that the observed network is not dense: the level of cooperation between the scientists in this research field seems to be low [36].

**Table 10:** Properties of the co-author network.

Sl	Description	Values
1	Nodes (# authors)	2581
2	Edges (# co-authorships)	6156
3	Average degree	4.7702
4	Density	0.0018

K-core is the largest sub graph of a certain co-author network where nodes have at least k (here k = 10) interconnections. Figure 3 provides a sub-graph of 25 out of 2581 nodes in Figure 2. These 25 authors have 10 or more co-author links during the study period.

### Prolific Authors

Table 11 provides the rank list of the top ten authors in the field. The ranks are based on publication numbers (frequency) and h*-*index. The top ten authors published between 19 and 60 papers during the study period. A total paper (TP) of authors shows the amount of publication credit of a concerned author which is obtained by fractional counting method. These authors were from the USA, Japan, Germany, China, and Switzerland. Bhushan B., who is also prominently visible in the co-authorship network of Figure 2, was the most prolific author, with 60 papers, and had the highest h-index of 28. Carpick R. W. ranked fifth in terms of paper numbers and had the second-highest h*-*index (h = 16) after Bhushan B.

**Table 11:** Prolific authors.

Author	Frequency (R)	TP	TC	h (R)
Bhushan B.	60 (1)	38.84	922.65	28 (1)
Wen S.	36 (2)	11.7	44.6	6 (5)
Fukuzawa K.	26 (3)	5.72	25.7	5 (6)
Zhang H.	26 (3)	5.61	21.57	4 (7)
Miyake S.	23 (4)	8.23	26.82	4 (7)
Mitsuya Y.	23 (4)	5.23	24.63	5 (6)
Schaefer J. A.	23 (4)	5.91	44.64	7 (4)
Ahmed S. I. U.	23 (4)	5.81	49.18	7 (4)
Scherge M.	19 (5)	5.92	90.95	11 (3)
Carpick R. W.	19 (5)	5.32	145.82	16 (2)

R = rank, TP = total papers, TC = total citations, h = h-index.

### Research Trend

The nanotribology research trends can be obtained by analysing the author keywords appended to the research papers across different time periods [37]. Word Cluster Analysis [12] is applied to analyse the author keywords. In this method, words which have plural forms, abbreviations and their transformations are grouped into single keywords. 860 papers (65%) out of all papers included author keywords. Analysis of keywords during the study period revealed that 1608 author keywords were used. Among them, 1193 (74%) keywords appeared once and 182 keywords appeared twice. The large number of keywords which appeared only once indicates that there was a lack of continuity in research [38]. Author keywords appended to the nanotribology research papers during 1996–2010 were ranked by total papers and five year block periods. Table 12 provides the frequency and rank of author keywords which appeared at least 22 times during the study period. Nanotribology/Microtribology, Atomic Force Microscope, Friction, Wear and Adhesion are the most popular keywords. Any one of the above keywords appeared in 951 papers (72%). 25% of all papers dealt with instruments like Atomic Force Microscope, Friction Force Microscope, and Scanning Probe Microscope. Nanoindentation is gaining popularity among the test methods. Friction, Wear, Lubrication and Adhesion are frequently used topics during the study period. Molecular Dynamics, MEMS, Hard Disk and Diamond like carbon are the only research topics in the keywords listed in Table 12 while the others are (nano)tribology related techniques and studies. Since these keywords moved up the ranks steadily from 1996–2000 to 2006–2010, they could be the major research topics in the future.

**Table 12:** Frequently used author keywords.

Keywords	TP	1996-10 R (%)	96-00 R (%)	01–05 R (%)	06–10 R (%)
Nanotribology/Microtribology	438	1 (33.2)	1 (31.2)	1 (28.6)	1 (36.6)
Atomic Force Microscope	198	2 (15.0)	2 (15.3)	2 (14.0)	2 (15.5)
Friction	166	3 (12.6)	3 (9.3)	3 (13.3)	3 (13.1)
Wear	82	4 (6.2)	4 (7.0)	4 (5.7)	4 (6.3)
Adhesion	67	5 (5.1)	5 (3.7)	8 (3.8)	5 (6.3)
Tribology	60	6 (4.5)	7 (2.8)	5 (5)	6 (4.8)
Diamond Like Carbon	54	7 (4.1)	11 (1.9)	6 (4.8)	7 (4.4)
Self-assembled Monolayers	44	8 (3.3)	32 (0.9)	10 (3.1)	8 (4.2)
Nanoindentation	43	8 (3.3)	19 (1.4)	7 (4.0)	10 (3.4)
MEMS	41	9 (3.1)	20 (1.4)	11 (2.9)	9 (3.8)
Molecular Dynamics	33	10 (2.5)	12 (1.9)	22 (1.4)	11 (3.4)
Boundary Lubrication	29	11 (2.2)	12 (1.9)	12 (2.6)	12 (2.0)
Thin Film	24	12 (1.8)	21 (1.4)	12 (2.6)	21 (1.5)
Hard Disk	23	13 (1.7)	62 (0.5)	15 (2.4)	18 (1.7)
Lubrication	22	14 (1.7)	8 (2.8)	18 (1.7)	23 (1.3)
Scanning Probe Microscope	22	14 (1.7)	14 (1.9)	9 (3.3)	61 (0.6)

TP = total papers, R = rank.

## Discussion

This study identified the leading countries, institutes, authors, core journals in the field of nanotribology research and examined their citation impact. Average number of authors per paper was 3.52 which is similar to research on Tsunami (3.1%) [39] and Bioinformatics (2.43) [40]. The 52% increase in collaboration of authors in nanotribology research can be seen as an expression of the field’s development towards ‘big science’ [41]. The share of non-cited papers is 30% in nanotribology research which is lower than in Acupuncture [42] where it was 38%. Moreover, Meho [43] estimated that around 90% of the papers published in academic journals are never cited. Papers with international collaboration in nanotribology research had more citation impact and increased visibility than national one. This result is in agreement with many other studies on other research fields. In nanotribology research, few authors (top 10 most productive) produced most of the papers (n = 278; 21%) and almost 12% of the most cited papers received 50% of all citations. This concentration of output and impact is also visible in many other research fields. The authors of highly cited papers in nanotribology research are not the same as the highly productive authors, which seems not to be consistent with Simonton’s model of creative productivity [44]. Core journals and journals publishing the highly cited papers in nanotribology research are from the subject areas of physics, chemistry, engineering, material science, biochemistry and computer science. This result shows the interdisciplinary nature of the research field.

Highly cited papers obtained through bibliometric analysis can be considered in collections of ‘suggested readings’ [45] which may provide the outline of particular research fields (here: nanotribology).

## Conclusion

The results of the present study on global nanotribology research output during 1996–2010 based on SCOPUS records explored key characteristics such as: growth rate of research, level of collaboration of publications, most publishing countries, most productive institutes, most preferred journals, citation impact, highly cited papers and research trend. The annual number of papers published grew tremendously from 34 in 1996 to 161 in 2010. Forty four countries engaged in nanotribology research during 1996–2010. Compared with other research fields like photosynthesis [17], where 156 countries were active, and nuclear waste management [46], where 140 countries were active, nanotribology seems to be a spatially concentrated research field. The USA published most of the (highly cited) papers. G7 countries contributed around 59% of the total papers. Tsinghua University, China, published the most papers. The top three journals were *Tribology Letters*, *Wear* and *Journal of Japanese Society of Tribologists*. Analysis of authorship pattern shows that co-authored publications dominate the research field. The visualization map indicates that the co-author network is not dense and only 25 authors (1%) have at least 10 co-author links during the study period. The results of this study could help the researchers (stakeholders) in the field of nanotribology as well as nanotechnology to understand the global research patterns and trends of nanotribology and to establish the future research directions.

There is one limitation of our study which should be addressed in future bibliometric studies on tribology or on other research topics. Since the nanotribology papers included in this study were attributes by Elsevier to several different subject areas in Scopus, field-normalized impact scores should actually be used to study research impact. The use of these scores is the standard in evaluative bibliometrics [47], in which for example different universities are compared based on papers attributed to different subject areas in a data base. We abstained from using normalized citation scores in this study because we used the bibliometric methods in a non-evaluative context of only a single research topic.

## References

[1] Krim J, Solina D H, Chiarello R (1991) Nanotribology of a Kr Monolayer: A Quartz-Crystal Microbalance Study of Atomic-Scale Friction. Phys Rev Lett 66: 181–184. 10043531 10.1103/PhysRevLett.66.181

[2] Jost H P (1996) *Lubrication (tribology) education and research*. A report on the present position and industry’s needs. Department of Education and Science, Her Majesty’s Stationery Office (London).

[3] Rensselar J V (2009) Nanotribology from lab to real world. Tribology & Lubrication Technology, 34–41.

[4] Gebeshuber I C, Macqueen M (2012) *Introducing the new Asian case method to Micro- and Nanotribology* Speech presented at the 6^th^ Intl. Coll. Micro- Tribology, Osieck-Warsaw, Poland.

[5] Carpick R W (2008) On the scientific and technological importance of nanotribology. Proceedings of the STLE/ASME International Joint Tribology Conference, Oct 2008, Florida, USA.

[6] Bhushan B (2008a) Nanotribology and nanomechanics in nano/biotechnology. Philos Trans A Math Phys Eng Sci 366 (1870): 1499–1537. 10.1098/rsta.2007.2170 18192166

[7] Bhushan B (2008b) Nanotribology, nanomechanics and nanomaterials characterization. Philos Trans A Math Phys Eng Sci 366 (1869): 1351–1381. 10.1098/rsta.2007.2163 18156126

[8] Rymuza Z (2010) Advanced techniques for nanotribological studies. Scientific Problems of Machines Operation and Maintenance, 1 (161): 33–43.

[9] Asha L G, Kumawal S C (2011) Nanotribology. International Journal of Advanced Engineering Technology, 2 (2): 300–310.

[10] 12^th^ US National Congress on Computational Mechanics website. Available: http://12.usnccm.org/ms8_4. Accessed 2013 Sep 02.

[11] Malarvizhi R, Wang M H, Ho Y S (2010) Research Trends in Adsorption Technologies for Dye Containing Wastewaters. World Applied Sciences Journal 8 (8): 930–942.

[12] Li J, Wang M H, Ho Y S (2011) Trends in research on global climate change: A Science Citation Index Expanded-based analysis. Glob Planet Change 77: 13–20.

[13] Modak J M, Giridhar M (2008) Scientometric analysis of chemical engineering publications. Current Science 94 (10): 1265–1272.

[14] Tsay M Y (2008) A bibliometric analysis of hydrogen energy literature, 1965–2005. Scientometrics, 75 (3): 421–438.

[15] Ortiz A P, Calo W A, Suárez-Balseiro C, Maura-Sardo M, Suárez E (2009) Bibliometric assessment of cancer research in Puerto Rico, 1903–2005. Pan Am J Public Healt, 25 (4): 353–361. 10.1590/s1020-49892009000400010 PMC3031111 19531324

[16] Sun J, Wang M H, Ho Y S (2012) A historical review and bibliometric analysis of research on estuary pollution. Mar Pollut Bull 66 (1): 13–21. 10.1016/j.marpolbul.2011.10.034 22119413

[17] Yu J J, Wang M H, Xu M, Ho Y S (2012) A bibliometric analysis of research papers published on photosynthesis: 1992–2009. Photosynthetica 50 (1): 5–14.

[18] Dong B, Xu G, Luo X, Cai Y, Gao W (2012) A bibliometric analysis of solar power research from 1991 to 2010. Scientometrics 93 (3): 1101–1117.

[19] Wang H, Liu M, Hong S, Zhuang Y (2013) A historical review and bibliometric analysis of GPS research from 1991–2010. Scientometrics 95 (1): 35–44.

[20] Cao Y, Zhou S, Wang G (2013) A bibliometric analysis of global laparoscopy research trends during 1997–2011. Scientometrics 96 (3): 717–730.

[21] Fu H Z, Wang M H, Ho Y S (2013) Mapping of drinking water research: A bibliometric analysis of research output during 1992–2011. Science of the Total Environment 443: 757–765. 23228721 10.1016/j.scitotenv.2012.11.061

[22] Kademani B S, Sagar A, Surwase G, Bhanumurthy K (2013) Publication trends in materials science: a global perspective. Scientometrics 94 (3): 1275–1295.

[23] Konur O (2011) The scientometric evaluation of the research on the algae and bio-energy. Applied Energy 88 (10): 3532–3540.

[24] Ball R, Tunger D (2006) Science indicators revisited – Science Citation Index versus SCOPUS: A bibliometric comparison of both citation databases. Information Services & Use 26: 293–301.

[25] Borsi B, Schubert A (2011) Agrifood research in Europe: A global perspective. Scientometrics 86 (1): 133–154.

[26] Hirsch J E (2005) An index to quantify an individual’s scientific research output. Proceedings of the National Academy of Sciences 102 (46): 16569–16572. 10.1073/pnas.0507655102 PMC1283832 16275915

[27] Ye F Y (2009) An investigation on mathematical models of the h-index. Scientometrics 81 (2): 493–498.

[28] Schubert A, Glänzel W (2007) A systematic analysis of Hirsch-type indices for journals. Journal of Informetrics 1 (2): 179–184.

[29] Egghe L, Rousseau R (2006) An informetric model for the Hirsch-index. Scientometrics 69 (1): 121–129.

[30] van Raan A F J (2006) Comparison of the Hirsch-Index with standard bibliometric indicators and with peer judgment for 147 chemistry research groups. Scientometrics 67 (3): 491–502.

[31] Schubert A (2007) Successive h-indices. Scientometrics 70 (1): 201–205.

[32] Borgatti S P, Everett M G, Freeman L C (2002) Ucinet for Windows: Software for Social Network Analysis. Analytic Technologies: Harvard, MA.

[33] Borgatti S P (2002) Netdraw network visualization. Analytic Technologies: Harvard, MA.

[34] Sci^2^ Team. (2009) Science of Science (Sci^2^) Tool. Indiana University and SciTech Strategies, http://sci2.cns.iu.edu.

[35] Adar E (2006) GUESS: A Language and Interface for Graph Exploration, CHI (http://graphexploration.cond.org).

[36] Erman N, Todorovski L (2011) Collaborative Network Analysis of two e-Government Conferences: Are we building a community? Electronic Journal of e-Government 9 (2): 141–151. Available at www.ejeg.com.

[37] Zhang G, Xie S, Ho Y S (2010) A bibliometric analysis of world volatile organic compounds research trends. Scientometrics 83: 477–492.

[38] Chuang K Y, Huang Y L, Ho Y S (2007) A bibliometric and citation analysis of stroke-related research in Taiwan. Scientometrics 72 (2): 201–212.

[39] Chiu W T, Ho Y S (2007) Bibliometric analysis of tsunami research. Scientometrics 73 (1): 3–17.

[40] Patra S K, Mishra S (2006) Bibliometric study of bioinformatics literature. Scientometrics 67 (3): 477–489.

[41] Glänzel W, Schubert A (2005) Analysing scientific networks through co-authorship. In Moed H F et al (eds), *Handbook of Quantitative Science and Technology Research*. Kluwer Academic Publishers, 257–276.

[42] Fu J Y, Zhang X, Zhao Y H, Tong H F, Chen D Z, et al (2012) Scientific production and citation impact: a bibliometric analysis in acupuncture over three decades. Scientometrics 93 (3): 1061–1079.

[43] Meho L I (2007) The rise and rise of citation analysis. Physics World, January, 32–36.

[44] Simonton D K (1997) Creative productivity: A predictive and explanatory model of career trajectories and landmarks. Psychological Review 104: 66–89.

[45] Lee J D, Cassano-Pinche A, Vincente K J (2005) Bibliometric analysis of human factors (1970–2000): A quantitative description of scientific impact. Human Factors 47 (4): 753–766. 10.1518/001872005775570970 16553064

[46] Kumar A, Girap P, Tewari S, Kademani B S, Bhanumurthy K (2011) Research Trends in Nuclear Waste Management: A Global Perspective. DESIDOC Journal of Library and Information Technology 31 (6): 452–459.

[47] Bornmann L, Marx W (2013) How good is research really? EMBO Reports 14 (3): 226–230. 10.1038/embor.2013.9 PMC3589094 23399654

